# DNA-PK Target Identification Reveals Novel Links between DNA Repair Signaling and Cytoskeletal Regulation

**DOI:** 10.1371/journal.pone.0080313

**Published:** 2013-11-25

**Authors:** Ewa Kotula, Wolfgang Faigle, Nathalie Berthault, Florent Dingli, Damarys Loew, Jian-Sheng Sun, Marie Dutreix, Maria Quanz

**Affiliations:** 1 Institut Curie, Centre National de Recherche Scientifique (CNRS) UMR3347, Institut National de la Santé et de Recherche Médicale (INSERM) U1021, Université Paris-Sud 11, Centre Universitaire, Orsay, France; 2 DNA Therapeutics, Evry, France; 3 Institut Curie, Centre de Recherche, Laboratory of Proteomic Mass Spectrometry, Paris, France; 4 University Hospital Zürich, Department of Clinical Neuroimmunology and MS Research, Paris, France; 5 Muséum National d’Histoire Naturelle, USM503, Paris, France; St. Georges University of London, United Kingdom

## Abstract

The DNA-dependent protein kinase (DNA-PK) may function as a key signaling kinase in various cellular pathways other than DNA repair. Using a two-dimensional gel electrophoresis approach and stable DNA double-strand break-mimicking molecules (Dbait32Hc) to activate DNA-PK in the nucleus and cytoplasm, we identified 26 proteins that were highly phosphorylated following DNA-PK activation. Most of these proteins are involved in protein stability and degradation, cell signaling and the cytoskeleton. We investigated the relationship between DNA-PK and the cytoskeleton and found that the intermediate filament (IF) vimentin was a target of DNA-PK *in vitro* and *in cells*. Vimentin was phosphorylated at Ser459, by DNA-PK, in cells transfected with Dbait32Hc. We produced specific antibodies and showed that Ser459-P-vimentin was mostly located at cell protrusions. In migratory cells, the vimentin phosphorylation induced by Dbait32Hc was associated with a lower cellular adhesion and migration capacity. Thus, this approach led to the identification of downstream cytoplasmic targets of DNA-PK and revealed a connection between DNA damage signaling and the cytoskeleton.

## Introduction

The genomes of all organisms are constantly being damaged by exogenous and endogenous agents. This has led to the evolution of a complex DNA damage signaling network that triggers DNA repair, cell cycle arrest or cell death in response to genotoxic treatments. DNA double-strand breaks (DSBs) are generally considered to be among the most toxic and mutagenic DNA lesions occurring in human cells. The response of mammalian cells to DSBs involves activation of the phosphatidylinositol–3 kinase–related kinases (PIKK) ataxia telangiectasia mutated (ATM), ataxia telangiectasia and Rad3-related (ATR), human SMG1 (hSMG) and DNA-dependent protein kinase (DNA-PK) [1]. ATM- and ATR-dependent signaling is mostly associated with DNA damage-induced cell cycle arrest and/or apoptosis. ATM can be activated by resected DSBs, in a complex with MRN and single-stranded DNA (ssDNA) [2] or directly, by reactive oxygen species [3]. ATR is mostly activated by the ssDNA ends of processed DSBs or collapsed replication forks [4]. The precise role of DNA-PK in damage signaling remains a matter of debate. DNA-PK, which comprises the DNA end-binding factor Ku, and the large catalytic subunit (DNA-PKcs), is activated when bound to double-stranded DNA and mediates efficient DSB repair through non homologous end joining (NHEJ) [5]. However, recent data have suggested that DNA-PK may play a much more important role in cellular signaling pathways than previously thought [6]. DNA-PK has been implicated in various nuclear and cytoplasmic signaling pathways, including EGFR signaling [7], [8] and NFκB signaling [9], [10], mRNA metabolism [11]–[13] and UV-induced metabolic gene activation [14].ATM, ATR and DNA-PK have similar substrate specificities *in vitro* (SQ/TQ), and partially overlapping substrate specificities *in vivo*. Recent studies have identified several hundred proteins containing the PIKK phosphorylation motif, the phosphorylation of which is induced in response to ionizing radiation (IR) [15], [16]. However, it is difficult to estimate the contribution of individual PIKKs, as IR induces various types of DNA lesion and also damages other cell components, leading to the activation of many signaling kinases [17]. We previously showed that 32 base pair (bp) short stabilized double-stranded DNA molecules (Dbait32Hc), which mimic DSBs, induce a specific DNA damage response in cells, with no chromosomal damage [18]. In the course of this response, which is dependent only on DNA-PK kinase activity, the DNA-PK targets become strongly and persistently phosphorylated, in both the nucleus and the cytoplasm [19]. Cell viability is not compromised as there is no chromatin damage. Dbait32Hc treatment is sensitizing tumors to radiotherapy and is currently under clinical investigation for local metastatic melanoma treatment (clinicaltrials.gov/show/NCT01469455). Therefore, it is particularly important to identify phosphorylated targets of DNA-PK after Dbait32Hc treatment as eventual activity biomarkers.

With the aim of identifying new downstream targets of DNA-PK, we used Dbait32Hc to investigate the impact of DNA-PK activation on the phosphoproteome, in a two-dimensional gel electrophoresis (2DE)-based approach. We observed an increase in the phosphorylation of proteins involved in cell signaling, protein stability and degradation and several proteins involved in the cytoskeleton. To our best knowledge a relationship between DNA-PK signaling and the cytoskeleton has never been described. We confirmed our observation by demonstrating the phosphorylation of the Ser459 residue of the intermediate filament (IF) vimentin by DNA-PK activity introduced in cells. These results show that a link exists between DNA damage signaling and the cytoskeleton.

## Materials and Methods

### Cell culture, irradiation, Dbait32Hc molecules, siRNA and transfection

Studies of cells in culture were performed with SV40-transformed MRC-5 (human fibroblasts, ATCC: CCL-171), ATM-defective AT5BI (human fibroblasts [20]), MCF-7 (human breast adenocarcinoma, ATCC: HTB-22), HCT116 (human colorectal carcinoma, ATCC: CCL-247), HeLa (human cervix adenocarcinoma, ATCC: CCL-2), M059K and DNA-PK-defective M059J (human glioblastoma, [21]) cells. Cells were grown at 37°C, as monolayers, in complete DMEM (Gibco, Cergy Pontoise, France) supplemented with 10% fetal calf serum (FCS), 110 mg/l sodium pyruvate and antibiotics (100 µg/ml streptomycin and 100 µg/ml penicillin) under conditions of 100% humidity, in an atmosphere of 95% air/5% CO_2_. SK-28 (human melanoma [22]) cells were grown in RPMI 1640 supplemented with 10% FCS and antibiotics. Cells were irradiated with the ^137^Cs unit of IBL-637 (ORIS, France) at room temperature (RT). Dbait32Hc molecules were produced by Eurogentec, as previously described [23]. The sequence of Dbait32Hc is 5’-GCTGTGCCCACAACCCAGCAAACAAGCCTAGA-(H)-TCTAGGCTTGTTTGCTGGGTTGTGGGCACAGC-3’ where H is a hexaethylene glycol linker. Cells were transfected with Dbait molecules in the presence of linear 11 kDa polyethyleneimine (PEI) (Polyplus-Transfection, Illkirch, France), according to the manufacturer’s instructions. Unless otherwise indicated, cells were transfected at 80% confluence, with 2 µg Dbait in 1.3 ml of culture medium without FCS (in 60 mm diameter plates) for 5 h. They were then left to recover for 1 h in medium supplemented with FCS. KU-55933 was purchased from Selleck Chemicals (Houston, TX, USA), and NU7026 and wortmannin were obtained from Sigma Aldrich (St. Louis, MO, USA). SiRNA specific for MOS (sc-39112, Santa Cruz Biotechnology, Inc. Germany) and control siRNA (ON-TARGETplus Non-targeting pool, Dharmacon) were then used to transfect the cells in the presence of DharmaFECT (Dharmacon), according to the manufacturer’s instructions.

### 2D gel electrophoresis

For 2DE, cells were treated with trypsin and washed 3 times with 250 mM sucrose in 10 mM Tris-HCl (pH 7.4). The pellets were resuspended in ∼80 µl/10^6^ cells 2D lysis buffer (7 M urea, 2 M thiourea, 0.5% 3/10 ampholytes (Bio-Rad, Marnes-la-Coquette, France), 4% (w/v) 3-(3-chloramidopropyl) dimethylammonio-1-propanesulfonate (CHAPS), 20 mM DTT), sonicated for 20 s on ice and incubated for 1 h at room temperature, with mixing by inversion. We added acrylamide to a final concentration of 60 mM acrylamide and the samples were incubated for 1 h at room temperature. Protein concentration was determined with the Bio-Rad Bradford protein assay and samples were stored at -80°C. Isoelectric focusing (IEF) was performed on 7 cm pH 3.0–10.0 (for analytical gels) or 24 cm pH 4.5–5.5 (for preparative gels) Immobiline DryStrips (GE Healthcare, Orsay, France). Before application, samples were diluted with 2D lysis buffer, with the concentration of the appropriate (3/10 or 4/7) ampholytes adjusted to 0.5% and trace amounts of bromophenol blue were added. We loaded 50 µg of sample for analytical gels and 300 to 350 µg for preparative gels, by passive rehydration. After IEF in a PROTEAN IEF Cell (Bio-Rad) for ∼52,900 Vh, the strips were allowed to equilibrate for 2×15 min with 100 mM Tris-HCl, pH 6.5, in 6 M urea, 30% glycerol and 2% SDS. DTT (1%) was included in the buffer for the first equilibration step and iodoacetamide (1%) was included in that for the second equilibration step, to reduce and alkylate free thiols. For the second dimension, 2D precast NuPAGE Bis-Tris 4–12% gradient minigels (Invitrogen, Cergy-Pontoise, France) were used for analytical electrophoresis and 1 mm×20 cm×24 cm 10–15% linear acrylamide gradient SDS gels were used for preparative electrophoresis. The IEF strips were placed on top of the gels and covered with 0.5% low-melting point agarose (containing methylcyanate as a blue stain) in SDS-PAGE running buffer. Preparative gels were run at ∼25 W per gel for ∼4 h or at ∼1 W per gel overnight, in the water-cooled Ettan DALTsix Large Vertical System (GE Healthcare). Gels were then fixed and stained with ProQ Diamond (Invitrogen), Sypro Ruby (Invitrogen) and SimplyBlue SafeStain (Invitrogen), according to the manufacturer’s instructions, and scanned after each staining step with a Typhoon Trio scanner (GE Healthcare), with the appropriate settings for each fluorophore. Further image analysis and quantification were performed with ImageMaster (GE Healthcare) or Melanie Viewer (Swiss Institute of Bioinformatics, Geneva, Switzerland) software.

### Trypsin digestion and mass spectrometry

In-gel digestion was performed as previously described [24], [25]. Briefly, excised gel slices were washed and the proteins they contained were reduced by adding DTT (Sigma Aldrich), before alkylation with iodoacetamide (Sigma Aldrich). The gel pieces were washed and shrunk by incubation with 100% acetonitrile. We then added trypsin (Sequencing Grade Modified, Roche Diagnostics) and incubated the gel slices overnight, for protein digestion. For phosphopeptide analysis, probes were directly used for nanoLC-MS/MS. The sample was first separated on a C18 reverse-phase column (75 µm i.d. x 15 cm, packed with C18 PepMap™, 3 µm, 100 Å; LC Packings), with a linear acetonitrile gradient (UltiMate 3000 system; Dionex), and MS and MS/MS spectra were then recorded on an LTQ Orbitrap XL™ mass spectrometer (Thermo Fisher Scientific, Villebon sur Yvette, France). The mass spectrometer was set to acquire a single MS scan followed by up to five data-dependent scans and, if a neutral loss of 98 Da from the precursor ion was observed in the CID mass spectrum, an MS3 scan of the neutral loss ion was also carried out (simultaneous fragmentation of neutral loss product and precursor, dynamic exclusion repeat count of 1, repeat duration of 30 seconds, exclusion duration of 180 seconds and lock-mass option enabled). The resulting spectra were then analyzed with Mascot™ and SEQUEST® Software (Matrix Science and Thermo Fisher Scientific), created with Proteome Discoverer (version: 1.2.0.92, Thermo Fisher Scientific), using the NCBInr *Homo sapiens (human* 2011 05 03, 236201 sequences) protein database. All phosphorylated peptides with unphosphorylated counterparts were validated by hand.

### *In vitro* phosphorylation and autoradiography

Vimentin protofilaments and filament complexes were reconstituted from purified vimentin (ab73843-10, Abcam, Cambridge, MA, USA) by stepwise drop dialysis (with “V” Series Membranes (0.025 µm pores, Millipore, Billerica, MA, USA)) of a 1 µg/µl vimentin solution in 9.5 M urea to 4 M urea (4 M urea, 30 mM Tris-HCl pH 7.4, 10 mM ammonium chloride, 2 mM EDTA, 2 mM DTT) then to physiological salt concentration (50 mM NaCl, 2 mM DTT, 10 mM Tris-HCl, pH 7.4) and finally to a DNA-PK activation buffer (20 mM KCl, 0.2 mM DTT, 10 mM HEPES/KOH pH 7.5, 2 mM MgCl_2_, 0.04 mM EGTA, 0.02 mM EDTA/KOH pH 7.5). Purified microtubules from mouse brain were kindly provided by Carsten Janke (Institut Curie, Orsay, France). The local Animal Experimentation Ethics Committee (Comité Ethique en Expérimentation Animale de l’Institut Curie, CEEA-IC, national registration number: #59) approved all animal experiments. We incubated 0–3.5 µg vimentin or 0–10 µg microtubules with 200 U purified DNA-PK (Promega, Lyons, France), 0.1 mM ATP, 0.001 mM (γ-^32^P)ATP (10 mCi/ml, Perkin Elmer, Courtaboeuf, France), 10 µg/ml bovine serum albumin (BSA) and 0.1 µg/µl Dbait32Hc or 0.03 µg/µl thymus DNA (Promega) in 20 mM KCl, 0.2 mM DTT, 10 mM HEPES/KOH pH 7.5, 2 mM MgCl_2_, 0.04 mM EGTA, 0.02 mM EDTA/KOH pH 7.5. The mixture was incubated for 30 min at 30°C and the reaction was stopped by adding 2×SDS sample buffer to give a final concentration of 50 mM Tris-HCl (pH 6.8), 1% β-mercaptoethanol, 2% SDS, 0.1% bromophenol blue and 10% glycerol. The denatured protein was separated by SDS-PAGE, in a 12% acrylamide/bisacrylamide (35.5/1) gel. The gel was dried and its storage Phosphor autoradiograph was scanned with a Storm 820 scanner (GE HealthCare) and analyzed with ImageQuant (GE HealthCare) software.

### Antibodies and immunological techniques

Rabbit polyclonal anti-Hsp90α-T5/7P and anti-vimentin-S56-P antibodies were purchased from Cell Signaling Technology (Danvers, MA, USA). Anti-DNA-PKcs-S2056P antibody was kindly provided by David. J. Chen (Department of Radiation Oncology, University of Texas Southwestern Medical Center, Dallas, TX, USA). The rabbit polyclonal anti-vimentin-S459-P antibody was produced and purified by BioGenes (Berlin, Germany) and was directed against the C-INET(pS)QHHDD peptide (BioGenes). Mouse monoclonal γ-H2AX antibody and the β-actin antibody AC-15 were obtained from Millipore and Sigma-Aldrich, respectively. Goat polyclonal anti-vimentin antibody (AB1620) was purchased from Millipore. Rabbit polyclonal anti-MOS (H-300) antibody was obtained from Santa Cruz Biotechnology, Inc, Germany.

For immunofluorescence staining, cells were processed as previously described [18]. Microscopy was performed at room temperature with the Leica SP5 confocal system, attached to a DMI6000 stand, with a 63×/1.4 oil immersion objective. Images were processed with the freely available ImageJ software (http://rsb.info.nih.gov.gate1.inist.fr/ij/) and the LOCI bioformat plug-in (http://www.loci.wisc.edu/ome/formats.html), to access images generated by the Leica SP5 confocal system. For immunoblotting, cells were lysed by scraping into Laemmli buffer and boiling for 10 min. The resulting lysates were then centrifuged and protein levels were normalized with the BCA protein assay kit (Thermo Scientific, USA). Proteins were separated by SDS-PAGE in 12% polyacrylamide (35.5 acrylamide/1 bisacrylamide) gels, transferred to nitrocellulose membranes, blocked by incubation with Odyssey buffer (LI-COR Biosciences, Lincoln, NE, USA) for 1 h and hybridized overnight at 4°C with primary antibody diluted in Odyssey buffer. For Western blots, the membranes were probed with goat anti-mouse or anti-rabbit secondary antibodies conjugated to Alexa Fluor 680 (Invitrogen) or IRdye 800 (Rockland Immunochemicals, Gilbertsville, PA, USA). The blots were imaged and quantified with the Odyssey Infrared Imaging System (LI-COR Biosciences, Lincoln) and Odyssey software. For detection of MOS protein the nitrocellulose membranes were blocked with 5% nonfat milk overnight at 4°C and hybridized also overnight at 4°C with primary antibody. Blots were then incubated with horseradish peroxidase conjugated goat anti-rabbit IgG secondary antibodies (sc-2004, Santa Cruz Biotechnology, Inc, Germany) and protein–antibody complexes were revealed on amersham hyperfilm (GE Healthcare, UK).

Cell cycle analysis was performed by flow cytometry, according to standard protocols. Briefly, the cells were fixed by incubation in ice-cold ethanol for 18 h, permeabilized by incubation with 0.2 % Triton X-100 for 15 min, treated with 25 U/ml RNAse A (Invitrogen) and DNA was stained with 50 µg/ml propidium iodide (Sigma-Aldrich). Cells were analyzed with a FACScalibur flow cytometer (BD Biosciences, Franklin Lakes, NJ, USA) and data were analyzed with the freely available WinMDI 2.8 (Scripps Research Institute, La Jolla, CA, USA) software.

### Wound healing assay and adhesion assay

Cells were cultured as monolayers in six-well plates until confluence and were then wounded with a yellow pipette tip. At least three wounds (in duplicate) were created for each set of experimental conditions, and the experiment was repeated two or three times. Wound closure was monitored in phase contrast mode, with a 4/5D microscope. This system consisted of a Leica DM IRBE microscope (Leica Microsystems, France) equipped with an incubation chamber (37°C, humidified atmosphere containing 5% CO_2_), an x–y–z stage controller and a charge-coupled device (CCD) CoolSNAP HQ2 camera (Photometrics, Tucson, AZ). Images were acquired at 15-minute intervals over a period of 16 h, with Metamorph software (Molecular Devices, Sunnyvale, CA). Movies were then reconstructed with the “nd stack builder”, plugins from ImageJ software (http://rsbweb.nih.gov/ij/plugins/track/builder.html) developed by F. Cordelières at Institut Curie (Orsay, France). Wound closure area was calculated manually with ImageJ software, as the percentage of the wound closed after 16 h of cell migration. Adhesion assays were carried out after transfection with Dbait32Hc or 8H by using a fibronectin-coated CytoSelect-48 cell adhesion assay (Cell Biolabs, San Diego, CA, USA) in accordance with the manufacturer’s protocol. Briefly, cells were counted and plated (1×10^5^ cells in serum-free medium/well) on fibronectin-coated plates then incubated for 60 min at 37°C in a cell culture incubator. After incubation, adherent cells were stained and quantified at OD560nm after extraction.

## Results

### Identification of 26 proteins displaying differential phosphorylation upon DNA-PK activation

**Figure 1:**
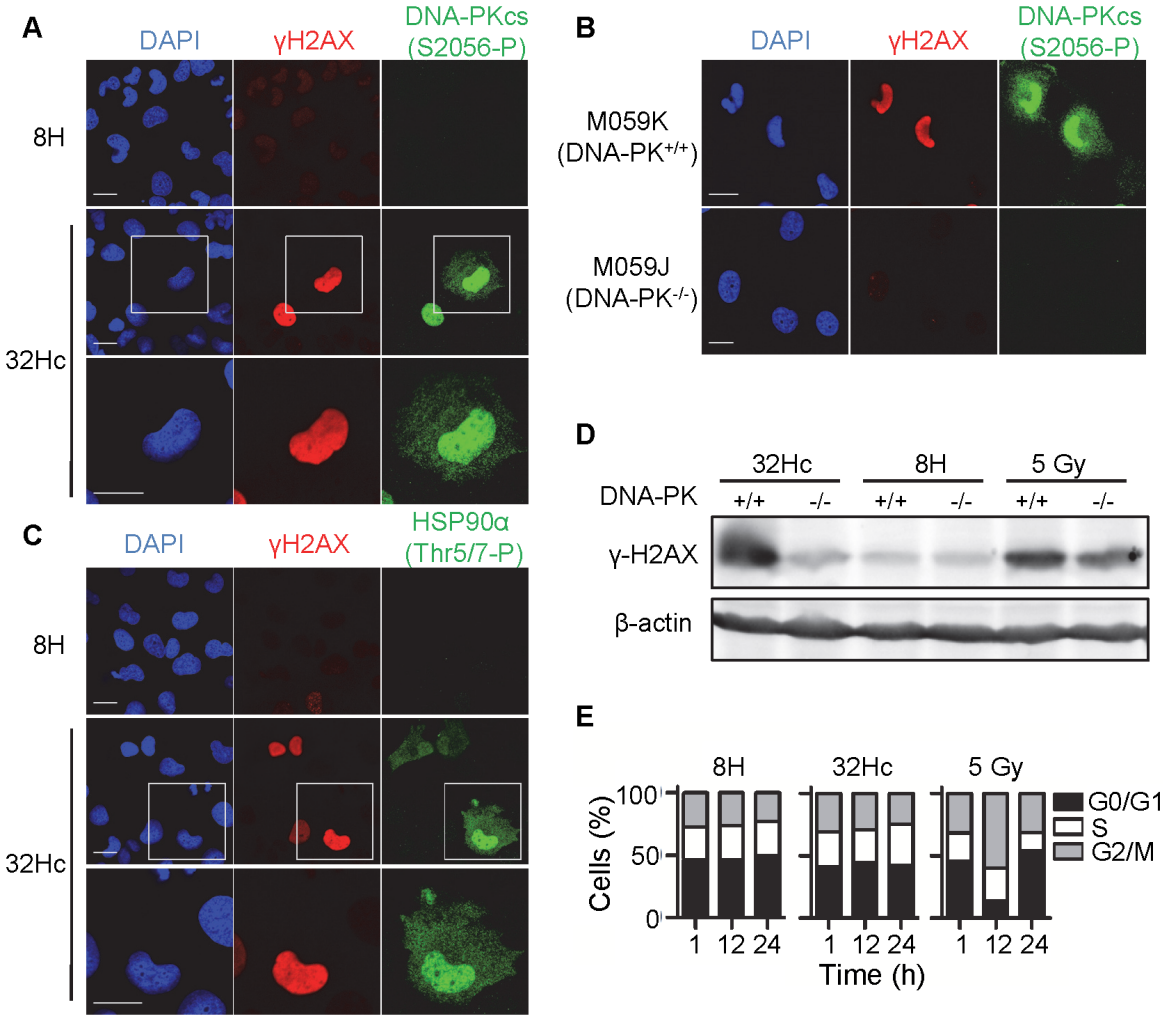
Nuclear and cytoplasmic activation of DNA-PK by Dbait32Hc. Immunostaining of DNA-PK (S2056-P) (green) and γ-H2AX (red) in 8H and Dbait32Hc-treated in (A) MRC-5 cells and (B) and MO59K (DNA-PK^+/+^) or DNA-PK-deficient M059J (DNA-PK^-/-^) cells. (C) Immunostaining of Hsp90α (Thr7-P) (green) and γ-H2AX (red) in in 8H and Dbait32Hc-treated MRC-5 cells. (A–C) Cells were fixed 1 h after the end of transfection with 8H or Dbait32Hc molecule. DNA was stained with DAPI (blue). Scale bar: 20 µm. (D) γ-H2AX activation in protein extracts 1h after the end of Dbait32Hc or 8H or γ-irradiated (5 Gy) treatment in M059K (DNA-PK^+/+^) and M059J (DNA-PK^-/-^) cells. The protein extracts were subjected to western blotting and the blots were probed with antibodies against γ-H2AX and β-actin. (E) Flow cytometric cell cycle analysis of 8H- or Dbait32Hc-transfected or irradiated (5 Gy) MRC-5 cells. Cells were fixed at the indicated times and DNA was stained with propidium iodide.

We aimed to identify proteins phosphorylated *de novo* upon nuclear and cytoplasmic DNA-PK activation. We transfected MRC-5 (transformed human fibroblasts) cells with Dbait32Hc or with the transfection control 8H, which is too short to bind and activate DNA-PK [18]. We first confirmed that DNA-PK was activated by Dbait32Hc molecules and not by the 8H control in MRC-5 cells. DNA-PK activation at the DNA double-strand break leads to autophosphorylation *in trans* at multiple sites [26]. Dbait32Hc treatment led to DNA-PK autophosphorylation at Ser2056 not only in the nucleus, as had already been shown after irradiation [26], but also in the cytoplasm in 40% of the transfected cells (Fig. 1A). The transfection control, 8H, did not induce DNA-PK autophosphorylation (Fig. 1A). DNA-PK activation by Dbait32Hc in both cytoplasm and the nucleus was further confirmed by the high levels of nuclear and cytoplasmic Hsp90α phosphorylation at Thr5/7, which we have recently show to be a cytoplasmic target of DNA-PK [19] (Fig. 1C). Consistently, the nuclear DNA-PK target, the histone H2AX, was strongly phosphorylated in response to Dbait32Hc. An analysis of the response in DNA-PK-deficient M059J glioma cells, based on a comparison with their DNA-PK proficient counterparts, M059K cells, confirmed the strict DNA-PK dependence of the Dbait32Hc-induced phosphorylation response (Fig. 1B, D). By contrast, irradiation, which leads to the activation of ATM and ATR in addition to DNA-PK, induced H2AX phosphorylation (γ-H2AX) in the DNA-PK-deficient cells (Fig. 1D). The selective activation of DNA-PK by Dbait32Hc, was not sufficient to induce a full DNA damage response (DDR), and thereby did not affect the cell cycle (Fig. 1E). Additionally activation of DNA-PK during apoptosis, which leads it’s autophosphorylation (S2056-P) and H2AX phosphorylation (γ-H2AX), does not result in vimentin phosphorylation on Ser459 (Figure S2 B).

**Figure 2:**
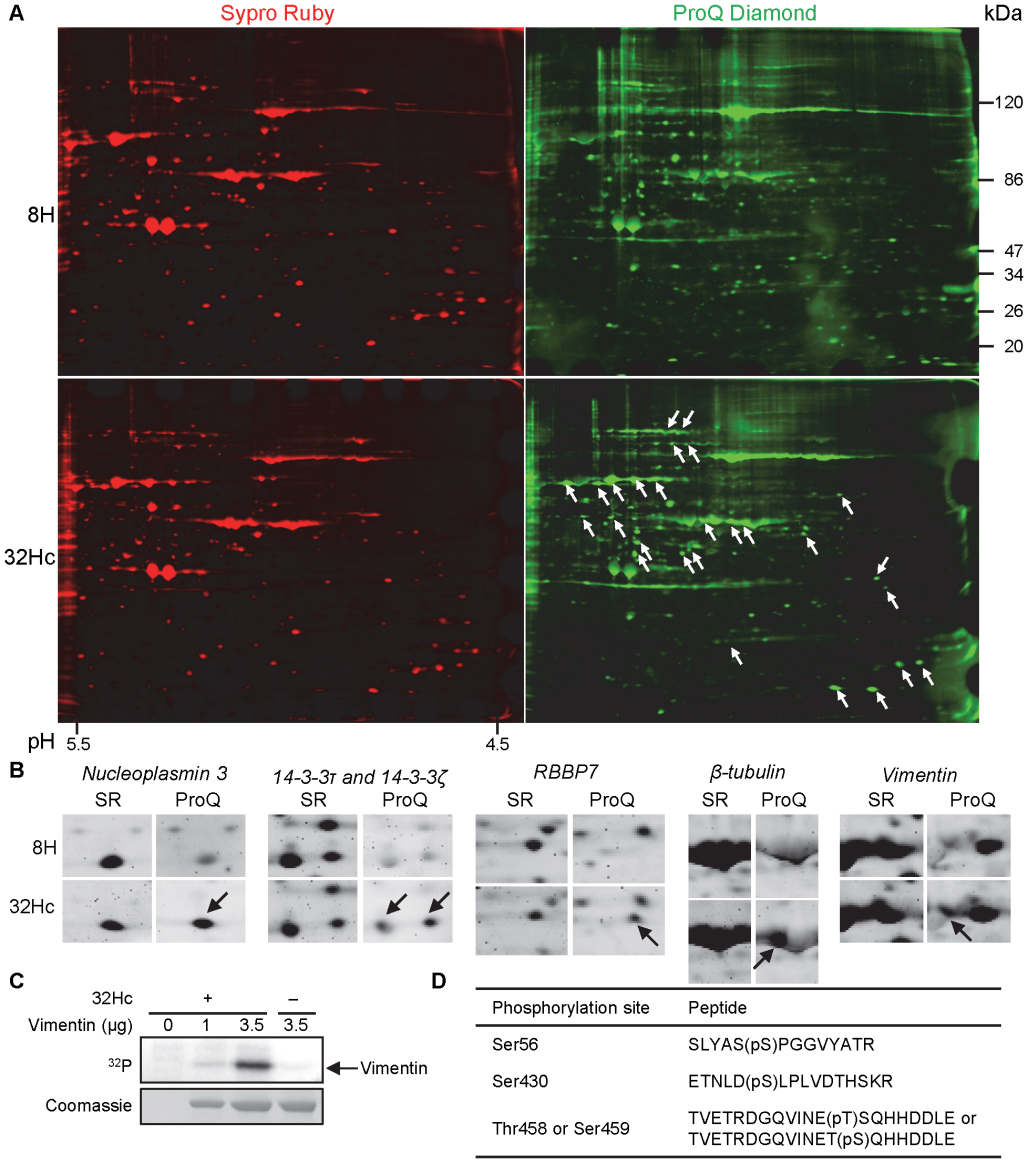
Differential phosphorylation pattern and total protein status after Dbait32Hc treatment. (A) SDS-polyacrylamide gel electrophoresis after isoelectric focusing (pH range 4.5–5.5) of MRC-5 lysates treated with Dbait32Hc or 8H. Total protein was detected by Sypro Ruby (red, SR) staining and phosphorylation was monitored by Pro-Q Diamond (green, Pro-Q) staining of the same gel. Spots displaying a marked increase in phosphorylation after treatment are highlighted (white arrows). (B) Higher magnification of selected spots from (A) showing higher levels of phosphorylation (arrows) of the indicated proteins after Dbait32Hc treatment than after transfection with the control, 8H. No difference in total protein levels was founds. Proteins displaying at least a 10-fold increase in Pro-Q Diamond staining that could be unambiguously assigned to proteins stained by Sypro Ruby were excised and analyzed by LC-MS/MS. (C) *In vitro* phosphorylation of vimentin by DNA-PK. Purified DNA-PK (DNA-PKcs and Ku) was incubated with [γ-^32^P]ATP and the indicated amounts of purified vimentin protein. Dbait32Hc was added where indicated, to activate DNA-PK, and the proteins were then denatured, separated by SDS-polyacrylamide gel electrophoresis and analyzed by autoradiography. (D) Peptides and phosphosites of *in vitro* DNA-PK-phosphorylated vimentin, as identified by LC-MS/MS with the LTQ-Orbitrap after trypsin digestion. (pT) and (pS) correspond to phosphorylated threonine and serine, respectively.

We identified proteins phosphorylated upon global DNA-PK activation, by subjecting the lysates of Dbait32Hc- or 8H-treated MRC-5 cells to 2DE. We analyzed a differential protein phosphorylation between the two conditions, using the fluorescent phosphoprotein dye Pro-Q Diamond (Pro-QD) which allows for detection of phosphate groups on serine, threonine or tyrosine residues. Total protein levels were monitored by staining with another fluorescent dye, Sypro Ruby. The greatest differences between the Dbait32Hc and 8H conditions were detected in a pH range between 4.5 and 5.5, which was therefore chosen for preparative 2DE (an example is shown in Fig. 2). The spots were detected and quantified and those displaying *de novo* phosphorylation after Dbait32Hc treatment or levels of phosphorylation after this treatment at least 10 times greater than those after 8H treatment, were excised and subjected to mass spectrometry (MS) analysis. No significant difference in total protein levels were observed between the two conditions, for any of the spots excised. Proteins identified unambiguously by MS analysis and corresponding to the isoelectric point (pI) and molecular weight (MW) of the excised spot are listed in Table 1 (more detailed MS data, including Mascot Score, are provided in Table S1). Changes in phosphorylation level upon DNA-PK activation by Dbait32Hc were observed for 26 proteins, 19 of which are also known to be present in the cytoplasm (Table S1), suggesting that DNA-PK may play an important role in cellular signaling via the phosphorylation of its targets in the cytoplasm. Phosphorylation levels increased for 25 of these proteins and decreased for the remaining protein, Tetratricopeptide repeat domain 1 (TPR1). TPR1 interacts with Ras [27] and modulates the activity of heat shock protein 70 (Hsp70) [28]. Two proteins, valosin containing protein (VCP) and heat shock protein 90α (Hsp90α), which we and others have already identified as direct targets of DNA-PK, were detected in this assay [19], [29], [30]. The other proteins phosphorylated after Dbait32Hc treatment had not previously been identified as DNA-PK targets.

**Table:** Table 1 Identified proteins phosphorylated upon DNA-PK activation.

*Protein*	*Gene name*	*Accession* a
14-3-3 protein τ	YWHAQ	P27348
14-3-3 protein ζ/δ	YWHAZ	P63104
DA41	UBQLN1	Q9UMX0
Desmoplakin isoform I	DSP	P15924
Eukaryotic translation initiation factor 4A isoform 1	EIF4A1	P60842
FK506-binding protein 4	FKBP4	Q02790
Heat shock 105kD	HSPH1	Q5TBM3
Heat shock 70kDa protein 1A variant	HSPA1A	P08107
Heat shock 70kDa protein 4	HSPA4	Q9BUK9
Heat shock 70kDa protein 8 isoform 1	HSPA8	Q53GZ6
Heat shock protein 90kDa α (cytosolic), class A member 1 isoform 1	HSP90AA1	P07900
Histone-binding protein RBBP7	RBBP7	Q16576
HMGCS1 protein	HMGCS1	Q01581
HnRNP F protein	HNRNPF	P52597
Lamin B1	LMNB1	P20700
Myosin, heavy polypeptide 9, non-muscle	MYH9	P35579
Mannose 6 phosphate receptor binding protein 1 (Perilipin-3)	PLIN3	O60664
NSFL1 (p97) cofactor (p47)	NSFL1C	Q9UNZ2
Nucleophosmin/Nucleoplasmin 3	NPM3	O75607
Ribosomal protein SA	RPSA	P08865
Tetratricopeptide repeat domain 1^b^	TTC1	Q99614
Tubulin, β	TUBB	P07437
Tubulin, β 2C	TUBB2C	P68371
Tubulin, β 6	TUBB6	Q9BUF5
Valosin-containing protein	VCP	P55072
Vimentin	VIM	P08670

a UniProtKB (www.uniprot.org).

b displayed loss of phosphorylation in response to Dbait32Hc treatment.

A large proportion of the proteins identified are involved in protein stability and degradation. In addition to TPR1, these proteins include VCP, nucleoplasmin 3, FK506-binding protein 4, DA41 (also known as ubiquilin 1), Hsp105, Hsp90α and several variants of Hsp70. Proteins involved in cell adhesion or related to the cytoskeleton formed another functional group among the identified proteins. This group includes ribosomal protein SA (also known as the 67 kD laminin receptor), vimentin, desmoplakin, myosin heavy polypeptide 9, lamin B1 and β-tubulin. The intermediate filament (IF) vimentin has been implicated in cancer invasion and metastasis [31]–[35]. High levels of vimentin are associated with a poor prognosis [34] and vimentin has been identified as a possible target for molecular cancer therapy [36]. To our knowledge vimentin has never been reported to be a target of DNA-PK. We therefore investigated its phosphorylation by DNA-PK.

### Phosphorylation of vimentin *in vitro* by DNA-PK

We confirmed that vimentin is a target of DNA-PK, by incubating purified vimentin filaments with DNA-PKcs, Ku, Dbait32Hc and [γ-^32^P] ATP followed by autoradiography. We found that DNA-PK phosphorylated vimentin (Fig. 2C). No phosphorylation signals were detected in the control in which vimentin was incubated with ATP in the absence of DNA-PK (Figure S2 D). Thus, the observed phosphorylation was the result of *de novo* vimentin phosphorylation by activated DNA-PK. We identified the sites phosphorylated by DNA-PK, by isolating the bands containing phosphorylated proteins and subjecting them to MS analysis. Vimentin was found to be phosphorylated at three sites (Fig. 2D, for spectra see Figs. S3, S4, S5): Ser56, Ser430 and Thr458 or Ser459 (it was not possible to distinguish between these two possibilities because of the proximity of these two amino acids), the best matching the consensus phosphorylation site of DNA-PK (SQ/TQ).

### DNA-PK-dependent vimentin phosphorylation in cells

Of the three sites that we found to be phosphorylated by DNA-PK *in vitro*, the sequence around Ser459 best matched the preferential phosphorylation motif of DNA-PK (SQ or TQ) [37], [38]. Though DNA-PK-dependent phosphorylation of an “S-hydrophobic” consensus motif, which would include the other two residues, has also been reported [12], [39], [40], we focused on vimentin phosphorylated site at Ser459. However, an increase in the phosphorylation of vimentin at Ser56 has been observed in response to radiomimetic treatment [17]. Having an antibody already available against this site we analyzed the phosphorylation of vimentin at this site in response to DNA-PK activation by Dbait32Hc and irradiation in cells. Ser56 was not phosphorylated after irradiation or Dbait32Hc treatment in interphase cells (Figure S1B), in which the phosphorylation of γ-H2AX confirmed that PIKKs were activated by the treatments. Vimentin phosphorylation at Ser56, probably by p21-activated kinase (PAK), has already been observed during mitosis and is thought to be involved in the reorganization of vimentin filaments (Tang *et al*., 2005; Ivaska *et al.*, 2007). Consistent with this hypothesis, we observed vimentin phosphorylation at Ser56 in mitotic cells, regardless of treatment (Figure S1B, arrow). The increase in Ser56 phosphorylation observed by Bensimon *et al.* [17] may therefore be due to cell cycle arrest following radiomimetic treatment, rather than DNA damage signaling by PIKKs.

**Figure 3:**
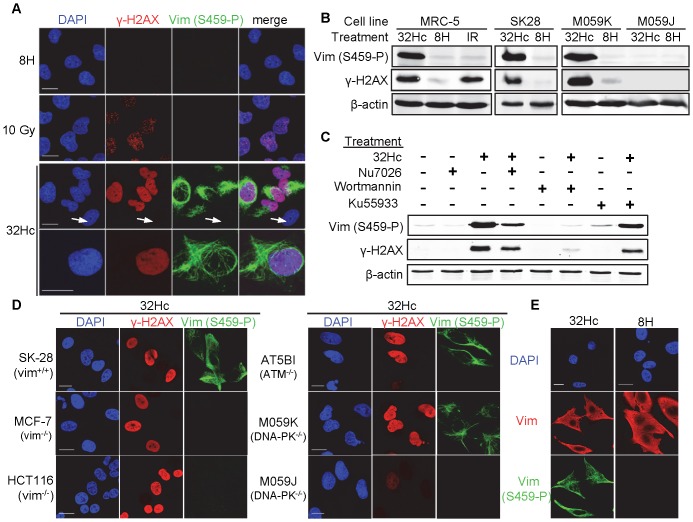
The Ser459 residue of vimentin is phosphorylated in response to Dbait32Hc. (A) Double immunostaining of anti-γ-H2AX (red) and anti-vimentin (S459-P) (green) in MRC-5 cells transfected with Dbait32Hc or 8H or irradiated (10Gy). The arrow indicates a cell negative for both γ-H2AX and phosphorylated vimentin (S459). (B) MRC-5, SK28, M059K or DNA-PK-deficient M059J cells were transfected with Dbait32Hc or 8H or irradiated (IR) with 10 Gy, as indicated. Proteins were extracted 1 h after the end of treatment for western blot analysis and the lysates were probed for vimentin (Vim-S459-P), γ-H2AX and β-actin. (C) MRC-5 cells were treated 2 h before and during Dbait32Hc or 8H treatment with 10 µM Nu7026 (DNA-PK inhibitor), 20 µM wortmannin (PIKK inhibitor), 10 µM KU-55933 (ATM inhibitor) or vehicle (DMSO). The total cell extracts were processed and probed as in (B). (D) The indicated cell lines were transfected with Dbait32Hc or 8H, fixed 1 h after the end of transfection and immunostained for vimentin (Vim-S459-P) (green) and γ-H2AX (red). (E) Double immunostaining of 32Hc- or 8H-treated SK28 cells with antibodies against total vimentin (Vim, red) and phosphorylated vimentin (Vim-S459-P, green). DNA was stained with DAPI (blue). Scale bar: 20 µm.

We then investigated Ser459 phosphorylation, by raising a polyclonal antibody against the phosphorylated site within the peptide INET(pS)QHHDD. This antibody detected high vimentin Ser459 phosphorylation in Dbait32Hc-treated cells, on both western blot and immunofluorescence analyses (Fig. 3). The specificity of this antibody was confirmed by immunoprecipitation and western blotting (Figure S1A). It detected no signal in Dbait32Hc-transfected MCF-7 breast adenocarcinoma cells, which express keratin IFs, but not vimentin IFs [41] as well as HCT116 colon cancer cell line lacking vimentin [35] (Fig. 3D). The observed phosphorylation signal was essentially cytoplasmic and displayed the characteristic filamentous structure of vimentin (Fig. 3A, D). About 70–90% of the cells treated with Dbait32Hc showed a high level of DNA-PK activation, detected by pan-nuclear γ-H2AX staining [18], and were all positive for vimentin phosphorylation at Ser459 (Fig. 3A, D). We observed no vimentin phosphorylation in response to IR, even at doses as high as 10 Gy, suggesting that vimentin phosphorylation might be specific for cytoplasmic DNA-PK activation by Dbait32Hc (Fig. 3A, B). Dbait32Hc-induced vimentin Ser459 phosphorylation was found in various human cell lines, including MRC-5 (transformed fibroblasts), SK28 (skin melanoma), AT5BI (ATM-deficient fibroblasts) and M059K (glioma) cells (Fig. 3A, D). Phosphorylation was dependent on DNA-PK, as it was completely abolished in DNA-PK-deficient M059J glioma cells (Fig. 3B and D) as well as in SK28 cells transformed by shDNA-PK plasmid (Figure S2A). Treatment with the selective DNA-PK inhibitor NU7026 strongly inhibited vimentin phosphorylation after Dbait32Hc treatment. ATM inhibition KU5593 leads to slight inhibitory effect what is due to not complete specificity to only ATM of this inhibitor. Treatment with the PI3K inhibitor wortmannin which inhibits both DNA-PK and ATM had the strongest inhibitory effect on vimentin phosphorylation (Fig. 3C). Dbait32Hc-induced phosphorylation did not affect total vimentin distribution (Fig. 3E).

**Figure 4:**
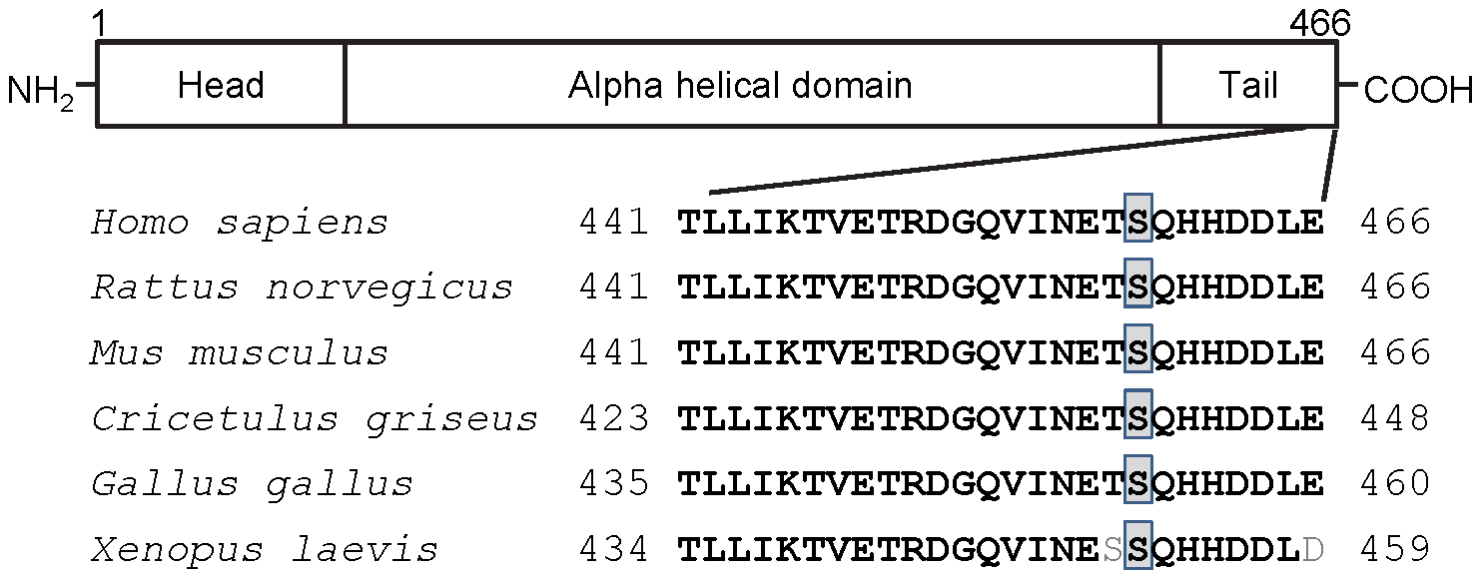
Diagram of the domain structure of vimentin and C-terminus conservation. Top, vimentin consists of a central α-helical rod domain, flanked by distal head (N-terminal) and tail (C-terminal) regions. Bottom, alignment of the sequence of the C-terminus of human vimentin (residues 441-466) with those of vimentin from various species. The human serine 459 residue is highlighted by gray boxes; residues differing between species are shown in light gray.

The Ser459 site, which is phosphorylated in a DNA-PK-dependent manner, is located at the extreme C-terminus of the protein (Fig. 4). The only function attributed to the C-terminus to date is *in vitro* interaction with F-actin [42]. The C-terminal sequence of vimentin is highly conserved across species, with 100% identity between humans (*H. sapiens*), rodents (*R. norvegicus*, *M. musculus*, *and C. griseus*) and chicken (*G. gallus*), and only two minor amino-acid differences (Thr → Ser and Glu → Asp) in frogs (*X. laevis*). We observed Dbait32Hc-induced vimentin phosphorylation on Ser459 in mouse embryonic fibroblasts (MEF, in 38% of transfected cells) or murine melanoma cells (B16BL6, in 50% of transfected cells) (Figure S1D). This high degree of conservation during evolution suggests that this phosphorylation site plays an important functional role.

### Phosphorylation of the vimentin Ser459 residue during mitosis

**Figure 5:**
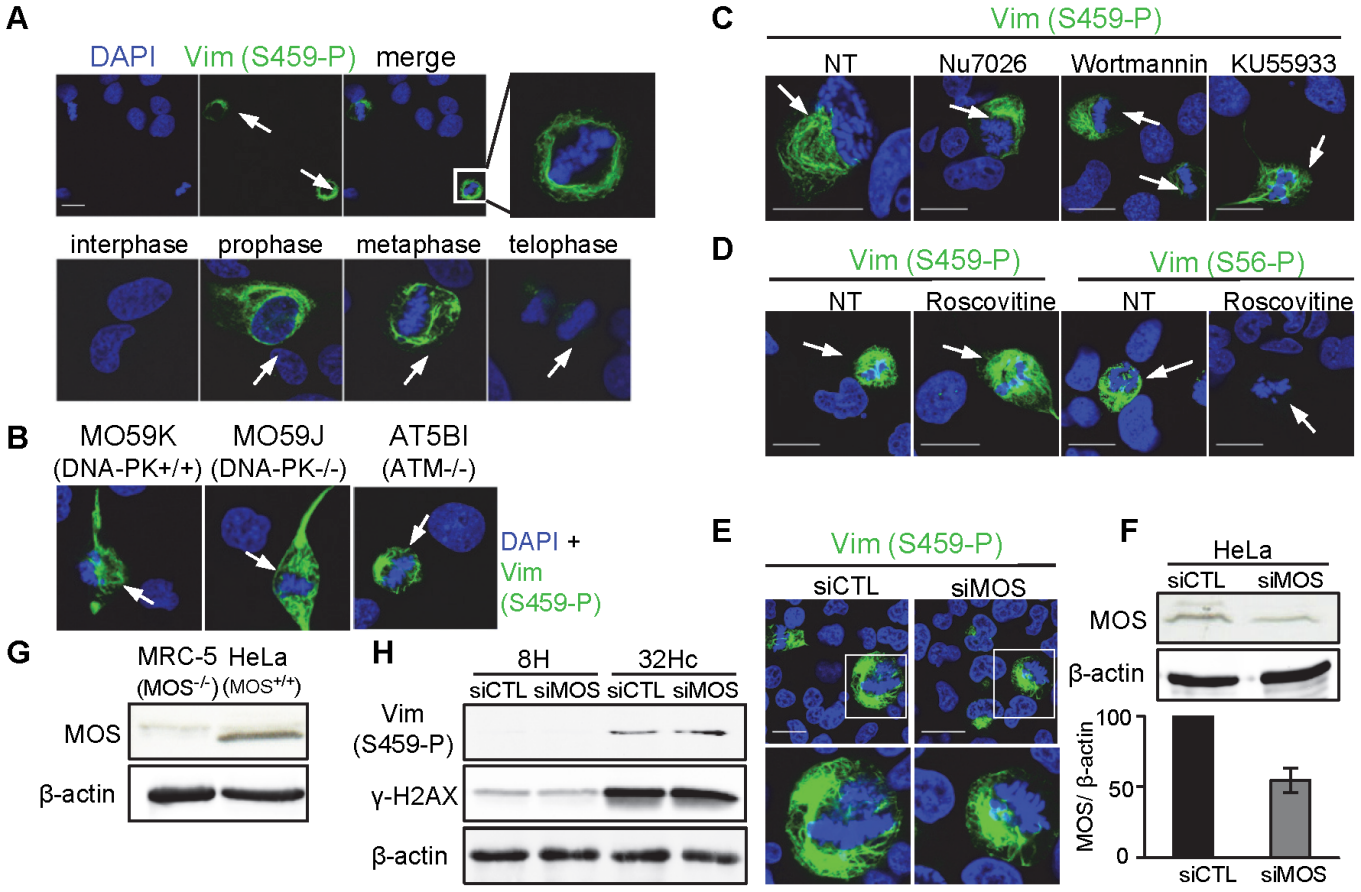
DNA-PK-independent phosphorylation of the Ser459 residue of vimentin in M-phase. (A) Merged images of immunostaining for vimentin (S459-P) (green) and DNA-staining with DAPI (blue) in untreated MRC-5 cells at the indicated stages of mitosis. (B) Immunostaining as in (A) of MO59K (DNA-PK^+/+^) or DNA-PK-deficient M059J (DNA-PK^-/-^) cells and ATM-defective AT5BI cells. (C) Immunostaining as in (A) of MRC-5 cells treated for 5 h with 10 µM NU7026 (DNA-PK inhibitor), 20 µM wortmannin (PIKK and PI3K inhibitor), 10 µM KU-55933 (ATM inhibitor) or (D) 60 µM roscovitine (CDK inhibitor). (E) MOS depletion does not affect vimentin phosphorylation at Ser459 during mitosis. HeLa cells were transfected with a control (CTL) or Mos-silencing siRNAs for 72h, followed by fixation and immunostaining as in A. (A-E) Cells in mitosis (arrow) or interphase are shown. Scale bar: 20 µm. (F) MOS depletion verification. HeLa cells were transfected with a control (CTL) or Mos-silencing siRNAs for 72h, followed by protein extraction, which were subjected to western blotting and the blots were probed with antibodies against MOS and β-actin. (G) MOS expression level in MRC-5 cells and HeLa cells. (H) MOS depletion does not affect Dbait32Hc-induced vimentin phosphorylation on Ser459. HeLa cells were transfected as described previously (E), 72h after transfection cells were treated with Dbait32Hc or 8H followed by proteins extraction, then subjected to western blotting and the blots were probed with vimentin (Vim-S459-P), γ-H2AX and β-actin.

We found that vimentin Ser459 was phosphorylated in untreated cells during the mitotic (M) phase of the cell cycle (Fig. 5A). The mitotic phosphorylation signal first became visible with chromatin condensation in early prophase and disappeared after anaphase, in early telophase, in all mitotic cells. Unlike the phosphorylation induced by Dbait32Hc, the mitotic phosphorylation of Ser459 was also observed in DNA-PK-deficient M059J cells (Fig. 5B) and in cells treated with the DNA-PK-specific inhibitor NU7026 (Fig. 5C). The phosphorylation of vimentin in M-phase is complex and has already been reported for Ser55, Ser56, Ser71, Ser72 and Ser82, which are phosphorylated by Cdk1 (cyclin-dependent kinase), CDK5, Rho kinase, Aurora B and Plk, respectively [43]-[46]. Vimentin has been shown to be phosphorylated on Ser459 by the p37mos protein kinase (MOS) during mitosis in oocytes [47], [48],[49]. Due to lack of any commercial antibodies against this phospho site these results were never completely confirmed. MOS (also called *c-MOS*) has been identified as the cellular homologue of v-mos oncogene, a product of Moloney murine sarcoma virus [50]. MOS is a proto-oncogene serine/threonine kinase that activates the MAP kinase cascade through direct phosphorylation of the MAP kinase activator MEK [51]. MOS plays an important role in oocyte maturation [52]. It is overexpressed in many tumors (lung carcinoma [53], [54]; gliomas [55], [56]) but not in non tumoral somatic cells. Its level is very low in transformed fibroblasts MRC5 (Fig. 5G, [57]), in which in our case we observe efficient vimentin phosphorylation on Ser 459 during mitosis (Fig. 5 A, C) and after Dbait32Hc treatment (Fig. 3A). Therefore it is very unlikely that MOS protein kinase is responsible for phosphorylation of vimentin on Ser459 at mitosis as well as after Dbait32Hc treatment. However, to eliminate this possibility we silenced MOS by siRNA in Hela cells which express MOS and monitored vimentin phosphorylation. Mitotic phosphorylation (Fig. 5E) as well as Dbait32Hc-induced phosphorylation (Fig. 5H) was not affected by MOS depletion (Fig. 5F). Mitotic phosphorylation at Ser459 was not dependent on ATM, another kinase involved in DNA damage signaling, as it was observed in both ATM-deficient AT5BI cells (Fig. 5B) and in cells treated with the ATM-specific inhibitor KU-55933 (Fig. 5C). PIKK kinases are known to have common targets and to be able to replace each other in some conditions. We therefore used wortmannin, an inhibitor with a low specificity, to inhibit all together ATM, ATR and DNA-PK, as well as PI3K [58]. Ser459 phosphorylation was still observed after wortmannin treatment (Fig. 5C), which efficiently reduced the activity of PIKK kinases, as shown by the low level of γ-H2AX formation after irradiation (Figure S1C). We also checked for an implication of cyclin-dependent kinases (CDKs) in the M-phase phosphorylation of this site by using roscovitine which inhibits CDKs 1,2,5,7 and 9 [59], [60]. While the CDK5-dependent phosphorylation of vimentin at Ser56 was suppressed, Ser459 phosphorylation was not affected (Fig. 5D). These findings suggest that none of kinase that belongs to neither the PIKK, the MOS nor the CDK family might be able to phosphorylate the Ser459 residue of vimentin in M-phase or these kinases can substitute each other in phosphorylating vimentin at mitosis.

### Impact of vimentin phosphorylation on migration and adhesion

**Figure 6:**
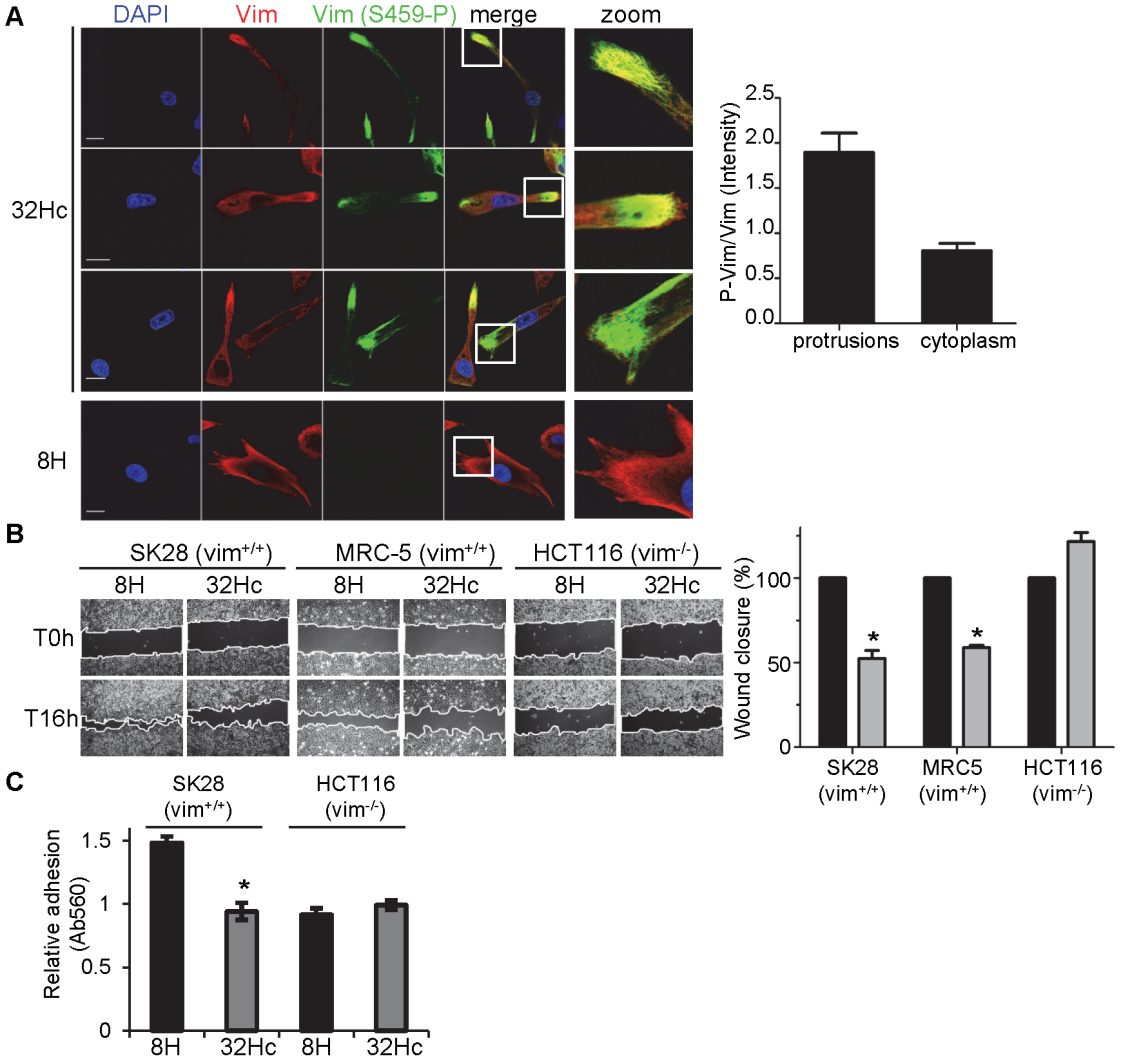
Inhibition of cell migration and adhesion by DNA-PK activation. (A) SK-28 cells were transfected with Dbait32Hc or control 8H, fixed 1 h after the end of transfection and immunostained for vimentin (red) and phospho-vimentin (S459-P) (green). A higher magnification of typical cell protrusions is shown. The bar chart provides a quantitative representation of the signal intensity ratio of vimentin (S459-P) and total vimentin for cell protrusions and cytoplasm, with standard deviations, from three analyses of 50 cells per set of conditions. DNA was stained with DAPI (blue). Scale bar: 20 µm. (B) The indicated cell lines were transfected with Dbait32Hc or 8H, wounded 1 h after the end of transfection and wound healing was observed by videomicroscopy for 16 h. The bar chart gives the percentage wound closure, with standard deviations, from three independent experiments. (C) The indicated cell lines were transfected with Dbait32Hc or 8H, then 1 h after the end of transfection cells were detached by trypsinization and let to adhere on fibronectin for 1h. The bar chart gives the relative adhesion to fibronectin measure by colorimetric assay, with standard deviations, from two independent experiments (Ab560). The significance of differences was assessed by the Kruskal-Wallis method. *, *p*<0.0005.

Vimentin is required for migration and invasion of cells [34], [35]. A closer examination of the vimentin phosphorylation pattern showed a preferential Ser459 phosphorylation in the cellular protrusions of melanoma cells (Fig. 6A). Quantification of the ratio of phosphorylated vimentin indicate that the vimentin is two-fold more phosphorylated when it is located in the protrusions than when it is cytoplasmic. Since cell protrusions are required for cell migration, we investigated the impact of the high vimentin phosphorylation in response to Dbait32Hc treatment on the cells’ capacity to migrate. In wound healing assay of MRC-5 and SK-28 cells, Dbait32Hc treatment led to diminished cell migration (Fig. 6B). We investigated the role of vimentin, by carrying out the same assay with HCT116 colon cancer cells, which do not express vimentin (Fig. 6C [35]) but are, nevertheless, invasive and able to migrate. The migration of vimentin-negative cells, unlike that of cells containing vimentin, was not inhibited by Dbait32Hc (Fig. 6B). Moreover, knowing that vimentin regulates cell adhesion [61], we investigated the role of vimentin phosphorylation after Dbait32Hc treatment on cell adhesion to fibronectin. Dbait32Hc treatment inhibits cell adhesion to fibronectin of SK-28 cells, but not of HCT116 cells lacking vimentin (Fig. 6C). These results strongly suggest that the phosphorylation of vimentin at Ser459 induced by DNA-PK regulate both cell migration and adhesion.

## Discussion

The DNA damage response is an intricate signaling network involving the phosphorylation of a multitude of proteins. Several studies [15]-[17] have shown that over 700 proteins are phosphorylated at PIKK consensus sites, in response to ionizing radiation or radiomimetic treatment. However, ionizing radiation induces a plethora of different DNA lesions and leads to the activation of various DNA damage signaling kinases. We therefore used DSB-mimicking Dbait32Hc molecules, which activate only DNA-PK, to investigate the contribution of this member of the PIKK family to the DNA damage response. Using 2DE to investigate proteins displaying *de novo* phosphorylation, in particular, we identified 25 proteins that were strongly phosphorylated in response to DNA-PK nuclear and cytoplasmic activation. These proteins included known DNA-PK substrates, the detection of which confirms that our approach can be used to identify new DNA-PK substrates. We chose a very stringent cutoff for spot selection, requiring not only at least a 10-fold increase in phosphorylation, but also a relatively high total protein spot intensity that could eventually be used as DNA-PK activity biomarker. Five of the proteins identified (FK506-binding protein 4, Heat shock 70 kDa protein 4, Heat shock 70 kDa protein 8, HnRNP F protein, and tubulin beta 6) were also reported to be phosphorylated in response to IR in the study by Matsuoka *et al.* [16]. We also identified two proteins of the 14-3-3 protein family, 14-3-3τ and 14-3-3ζ, that were phosphorylated upon DNA-PK activation by Dbait32Hc. 14-3-3 proteins are adaptor proteins that bind to a large number of partners and are involved in various cellular processes, including kinase regulation, adhesion, motility and nucleocytoplasmic transport [62], [63]. Both 14-3-3τ and 14-3-3ζ can bind and inhibit Cdc25C [64] and play a role in mediating the G2/M cell cycle checkpoints after DNA damage [65]. The phosphorylation of these proteins in our study suggests that they may be phosphorylated by DNA-PK in response to DNA damage.

Six of the proteins identified (ribosomal protein SA, vimentin, desmoplakin, β-tubulin, myosin heavy polypeptide, 9 and lamin B1) have functions in cell adhesion and the cytoskeleton. We investigated the DNA-PK-dependent phosphorylation of vimentin, as this major IF of mesenchymal cells plays a role in diverse cellular pathways and processes, including cell adhesion [61], [66], [67], migration [68], [69], infection [70], [71], ERK signaling [72], regulation of transcription [73] and the modulation of 14-3-3 protein-dependent signaling [74], [75]. Vimentin has also been implicated in cancer cell invasion and high levels of this protein are associated with poor treatment outcome in patients with cancer [32]–[35]. Moreover, multiphosphorylated vimentin has been described to be associated to invasiveness in meningiomas, highlighting a potential role of vimentin phosphorylation in invasion [76]. Consistent with our findings from the phosphoproteomic analysis, we demonstrated that DNA-PK phosphorylated purified vimentin *in vitro* and in cells. The phosphorylation pattern of vimentin is highly complex [61], [77]. Functions have been attributed to the phosphorylation of several residues towards the N-terminus of the protein, whereas no partner or function has yet been attributed to the C-terminus, which houses the Ser459 residue shown here to be phosphorylated by DNA-PK. The C-terminus is highly conserved and would therefore be expected to have important regulatory functions.

In the course of studying the phosphorylation of vimentin after Dbait32Hc treatment we observed that Ser459 was highly phosphorylated at mitosis in all the cells expressing vimentin. This phosphorylation seems to be independent of DNA-PK activity since inhibitors and mutations that suppress Dbait32Hc-induced Ser459 phosphorylation had no effect on mitotic phosphorylation. This result is coherent with the observation that Dbait32Hc treatment has no effect on cell cycle and do not affect proliferation [18]. We investigated PIKK, MOS and the CDK kinases family as possible kinases but none of the candidates were responsible for this mitotic phosphorylation. Up to date, all the cells expressing vimentin show phosphorylation of the Ser459 at mitosis and further studies are necessary to identify the kinase involved. We tried to produce vimentin with a mutation at Ser459 for preventing its phosphorylation, but it was not possible to create endogenous human vimentin network in cell lacking vimentin by expressing the protein from plasmid [41]. Moreover, overexpressing vimentin cDNA from plasmid in wild type background (cells expressing vimentin, SK28, data not shown) leads to endogenous vimentin networks disassembly, preventing further studies with Ser459 mutants. No interaction of vimentin with DNA-PK has ever been demonstrated. However, the production of vimentin has been reported to be regulated by poly (ADP-ribose) polymerase 1 (PARP-1) [78], another important DNA repair enzyme. Moreover, vimentin may be directly involved in DNA metabolism, because it has been shown to bind directly to DNA [79], [80] and to have an affinity for various DNA structures, including four-way junctions, G-quadruplexes [81] and triplex-DNA [82]. Vimentin is present in nuclear matrix attachment regions (MARs) [83], [84], which contribute to the structural organization of chromatin and seem to be involved in recombination and repair events. MARs seem to play a particularly important role in NHEJ, as the DNA-ligase IV/XRCC4 complex localizes to these regions and DNA-PK has been shown to redirect DNA ends to these sequences [85]. The absence of a significant increase in vimentin Ser459 phosphorylation in response to IR suggests that the signal may be locally restricted and transient.

The functional significance of cytosolic DNA-PKcs remains unclear. However, recent findings suggest that, in addition to its well established role in DNA repair in the nucleus, DNA-PK phosphorylates targets involved in various cellular signaling pathways, such as UV-induced translational reprogramming [14], NFκB activation [9], [10], mRNA metabolism [11]–[13], EGFR signaling [7], [8] and the regulation of Akt/PKB [86], [87]. NFκB has been demonstrated to be sequestered in an inactive state in the cytoplasm, when bound to IκB inhibitor family members [88]. The authors revealed that DNA-PK can directly phosphorylate IκB-β. It has been shown that DNA-PK is directly involved in NFκB activation in response to IR via the modification of IκB [9], [10]. As the NFκB- IκB inhibitor complex is localized in the cytoplasm, the signaling of DNA damage in the NFκB pathway might be an example of the essential cytoplasmic role of DNA-PK. Moreover, it has been identified that DNA-PK can also activate Akt/PKB [86], [87]. DNA-PKcs was shown to be colocalized with Akt at the plasma membrane and phosphorylates Akt on Ser473 resulting in a 10-fold enhancement of Akt activity. Other studies have demonstrated an interaction between EGFR and DNA-PK [89]. The EGFR-DNA-PK complex is generated in the cytoplasm and the inhibition of radiation-induced EGFR nuclear import leads to suppressed DNA-PK activity [7], [8]. These results suggest that one consequence of IR might be the internalization of EGFR together with DNA-PK present in the lipid rafts [90] or the cytoplasm and the nuclear translocation of the complex. Therefore, all these results suggest a cytoplasmic response to DNA damage transmitted by activated DNA-PK.

The vimentin network spreads from the nucleus to the plasma membrane and is involved in cell signaling, adhesion, migration and invasion processes [61]. We show here that Dbait32Hc-induced DNA-PK activation leads to the preferential phosphorylation of vimentin in cell protrusions. Dbait32Hc treatment also significantly decreased the capacity of cells to migrate. Dbait32Hc treatment induced a large set of protein modifications through DNA-PK activation, but the inhibition of cell migration seems to be directly linked to vimentin modification, because the migration of cells devoid of vimentin was not affected by Dbait32Hc treatment. Dbait32Hc may inhibit migration by modifying cytoskeleton dynamics, through the high level of phosphorylation of vimentin and, possibly, other cytoskeleton components, suggesting possible crosstalk between DNA-PK signaling and cytoskeletal functions, such as cell adhesion and migration. Moreover, DNA-PK has been reported to be overexpressed in various metastatic tumors [91] however the mechanism of its role in this process is not known yet. Here, we revealed a link between DNA-PK signaling, cytoskeleton and cell migration. Therefore, our results are very important for further understanding of the role of DNA-PK in cancer metastasis process.

The phosphorylation of vimentin could also play a role in viral infection, where the viral nucleic acids may be recognized by Ku and DNA-PK in the cytoplasm [92], [93]. Several results suggest that vimentin could play a role in viral infection. Vimentin has been shown to interact with the CPMV plant virus which is structurally related to animal picornavirus including the poliovirus [94]. Vimentin was reported to be phosphorylated at Ser82 and rearranged after infection of African swine fever virus infection [70]. Detecting nucleic acids in cytoplasm is an important part of recognizing pathogens and viral infection. This task seems to be assume by the DNA-PK enzyme which triggers via IRF-3 a sequence of events that lead to the innate immune response to be activated [95]. We propose that beside the control of the immune response DNA-PK could play another anti-viral activity by modifying vimentin.

In summary, we have shown that stabilized DSB-mimicking Dbait32Hc molecules may be used as a tool for studying DNA-PK-dependent signaling in cells. In this study, we demonstrated previously unknown target of DNA-PK which is IF vimentin. We mapped the DNA-PK phosphorylation sites of vimentin and confirmed the DNA-PK-dependent phosphorylation of vimentin at Ser459 in cells. We showed that Ser459-P-vimentin was mostly located at cell protrusions of migratory cells. We then demonstrated that vimentin phosphorylation at Ser459 induced by Dbait32Hc treatment leads to inhibition of cell adhesion and migration. Dbait32Hc treatment is sensitizing tumors to radiotherapy and is currently under clinical investigation for local metastatic melanoma treatment (clinicaltrials.gov/show/NCT01469455). Therefore, our results are very important as they provide evidence that Dbait32Hc treatment by inhibition of cancer cell migration can have anti-metastatic effect. Moreover, our results highlight the links between DNA-PK signaling, the cytoskeleton, cell adhesion and migration. These findings provide new insight into very important cellular processes controlled by DNA-PK.

## Supporting Information

Figure S1 **(A) Western blot data demonstrating the specificity of the anti-vimentin (S459-P) antibody.** (B) Immunofluorescence staining that shows no vimentin phosphorylation on Ser56 in response to DNA-PK activation by Dbait32Hc and irradiation in cells. (C) Control experiments demonstrating the inhibition of PIKKs by specific inhibitors. (D) Immunofluorescence staining of vimentin phosphorylation on Ser459 in response to DNA-PK activation by Dbait32Hc treatment in mouse cells (MEF’s - mouse embryonic fibroblasts, B16BL6 - mouse melanoma).
(PDF)

Figure S2 (**A) Immunofluorescence staining that shows no vimentin phosphorylation on Ser459 in response to DNA-PK activation by the Dbait32Hc molecule in SK28 cells transformed by shDNA-PK plasmid compared to cells transformed with the control (shCTL) plasmid. (B, C) Immunofluorescence staining that shows no vimentin phosphorylation on Ser459 in response to DNA-PK activation during apoptosis.** (D) *In vitro* phosphorylation of vimentin by DNA-PK.
(PDF)

Figure S3 **MS/MS spectrum for identification of vimentin** ***in vitro*** **phosphorylation site Ser459.**
(PDF)

Figure S4 **MS/MS spectrum for identification of vimentin** ***in vitro*** **phosphorylation site Ser56.**
(PDF)

Figure S5 **MS/MS spectrum for identification of vimentin** ***in vitro*** **phosphorylation site Ser430.**
(PDF)

Table S1 **Detailed MS data including the Mascot Scores of the proteins identified in the 2D gel experiment.**
(PDF)
