# Supplementary material for: DNA-PK Target Identification Reveals Novel Links between DNA Repair Signaling and Cytoskeletal Regulation
Source: PLoS One. 2013 Nov 25;8(11):e80313. doi: 10.1371/journal.pone.0080313 (PMC3840018; doi:10.1371/journal.pone.0080313)
Supplement: Figure S1 — (A) Western blot data demonstrating the specificity of the anti-vimentin (S459-P) antibody. (B) Immunofluorescence staining that shows no vimentin phosphorylation on Ser56 in response to DNA-PK activation by Dbait32Hc and irradiation in cells. (C) Control experiments demonstrating the inhibition of PIKKs by specific inhibitors. (D) Immunofluorescence staining of vimentin phosphorylation on Ser459 in response to DNA-PK activation by Dbait32Hc treatment in mouse cells (MEF’s - mouse embryonic fibroblasts, B16BL6 - mouse melanoma). (PDF) [file pone.0080313.s001.pdf]

FIGURE S1

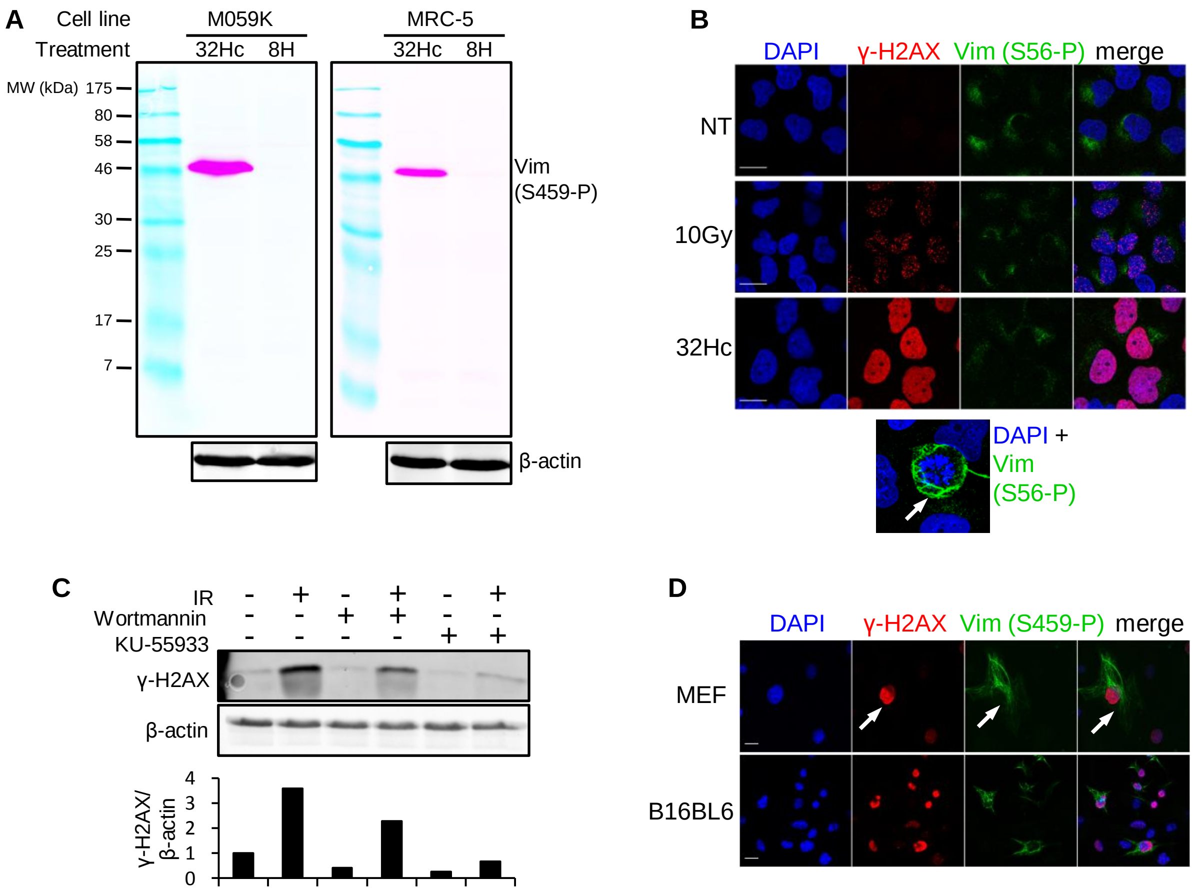

FIGURE S1. Specificity of anti-vimentin (S459-P) antibody, no vimentin phosphorylation on Ser56 in response to DNA-PK activation, controls for PIKK inhibitors and vimentin phosphorylation on Ser459 in murine cells. (A) M059K (left panel) or MRC-5 (right panel) cells were treated with 32Hc or the negative control 8H and lysed 1 h after the end of transfection. The total cell extracts were separated by SDS-PAGE, blotted and probed for vimentin (S459-P) and β-actin. Antibody binding was detected with fluorescent secondary antibodies, on an Odyssey infrared imager. Light blue corresponds to the molecular weight marker and violet to vimentin (S459-P). The two signals were merged. (B) Dbait32Hc-induced DNA-PK activation doesn't result in vimentin phosphorylation on Ser56. MRC-5 cells were transfected with Dbait 32Hc, irradiated (10 Gy) or left untreated (NT) and fixed 1 h after the end of transfection or irradiation. Double immunostaining was performed with anti-γ-H2AX (red) and anti-vimentin (S56-P) (green) antibodies. Arrow: cell undergoing mitosis. (C) MRC-5 cells were treated 5 h before irradiation (IR) with 20 µM wortmannin (PIKK inhibitor), 10 µM KU-55933 (ATM inhibitor) or vehicle (DMSO). The cells were irradiated (IR) with 10 Gy, then lysed, and extracts were processed as in (A) and probed for γ-H2AX and β-actin. The bar chart provides a quantitative representation of the western blot signal ratio for γ-H2AX to β-actin. (D) Immunofluorescence staining of vimentin phosphorylation on Ser459 (green) and anti-γ-H2AX (red) in response to DNA-PK activation by Dbait32Hc treatment in mouse cells (MEF's - mouse embryonic fibroblasts, B16BL6 - mouse melanoma). (C-D) DNA was stained with DAPI (blue). Scale bar: 20 µm
