# Supplementary material for: DNA-PK Target Identification Reveals Novel Links between DNA Repair Signaling and Cytoskeletal Regulation
Source: PLoS One. 2013 Nov 25;8(11):e80313. doi: 10.1371/journal.pone.0080313 (PMC3840018; doi:10.1371/journal.pone.0080313)
Supplement: Figure S2 — (A) Immunofluorescence staining that shows no vimentin phosphorylation on Ser459 in response to DNA-PK activation by the Dbait32Hc molecule in SK28 cells transformed by shDNA-PK plasmid compared to cells transformed with the control (shCTL) plasmid. (B, C) Immunofluorescence staining that shows no vimentin phosphorylation on Ser459 in response to DNA-PK activation during apoptosis. (D) In vitro phosphorylation of vimentin by DNA-PK. (PDF) [file pone.0080313.s002.pdf]

FIGURE S2

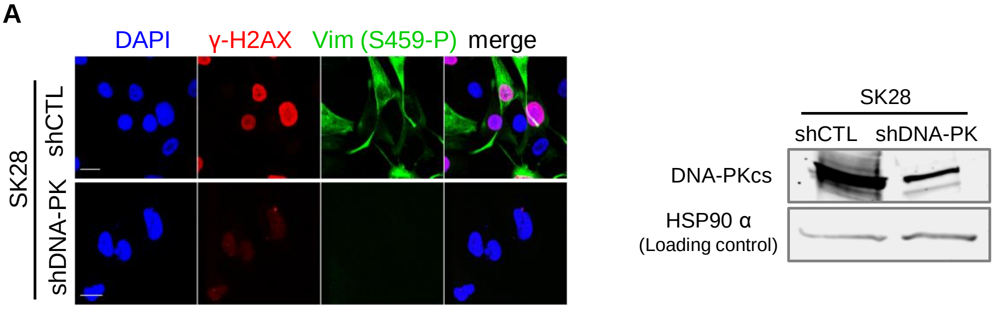

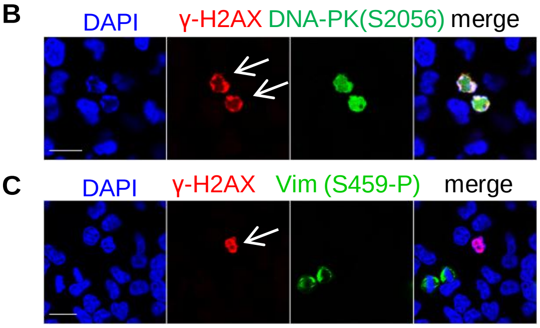

D

Peptide sequence	Modification	Score	Treatment
SLYAS(pS)PGGVYATR	**Phospho (Ser56)**	44	Vimentin +ATP +Dbait32c + DNA-PK
	Acetyl (N-term)	57	
	-	79.5	
	Acetyl (N-term)	68	Vimentin +ATP
	-	86	
ETNLD(pS)LPLVDTHSK	**Phospho (Ser459)**	21	Vimentin +ATP +Dbait32c + DNA-PK
	Acetyl (N-term)	56	
	-	67	
	Acetyl (N-term)	52	Vimentin +ATP
	-	64	

FIGURE S2. No vimentin phosphorylation on Ser459 in response to DNA-PK activation by Dbait32Hc molecule in SK28 cells transformed by shDNA-PK plasmid, no vimentin phosphorylation on Ser459 after DNA-PK activation in apoptotic cells and vimentin phosporylation *in vitro* by DNA-PK. (A) Double immunostaining of anti-γ-H2AX (red) and anti-vimentin (S459-P) (green) in SK28 cells transformed by control (shCTL) or shDNA-PK plasmid. Proteins of SK28 cells stably transformed by control (shCTL) or shDNA-PK plasmid were extracted for western blot analysis and the lysates were probed for DNA-PK for silencing veryfication and HSP90α as an loading control. (B, C) HeLa cells were treated with TRAIL Killer 100ng/ml during 8h to induce apoptosis, then double immunostaining was performed of (B) anti-γ-H2AX and anti-DNA-PK (S2056-P) (green) and (C) anti-γ-H2AX (red) and anti-vimentin (S459-P) (green). Staining of anti-γ-H2AX (red) was used as positive control for apoptotic rings detection (narrows). DNA was stained with DAPI (blue). Scale bar: 20 µm. (D) Peptides and phosphosites of *in vitro* DNA-PK-phosphorylated vimentin, as identified by LC-MS/MS with the LTQ-Orbitrap after trypsin digestion. (pS) correspond to phosphorylated serine.
