# Supplementary material for: DNA-PK Target Identification Reveals Novel Links between DNA Repair Signaling and Cytoskeletal Regulation
Source: PLoS One. 2013 Nov 25;8(11):e80313. doi: 10.1371/journal.pone.0080313 (PMC3840018; doi:10.1371/journal.pone.0080313)
Supplement: Figure S3 — MS/MS spectrum for identification of vimentin in vitro phosphorylation site Ser459. (PDF) [file pone.0080313.s003.pdf]

FIGURE S3

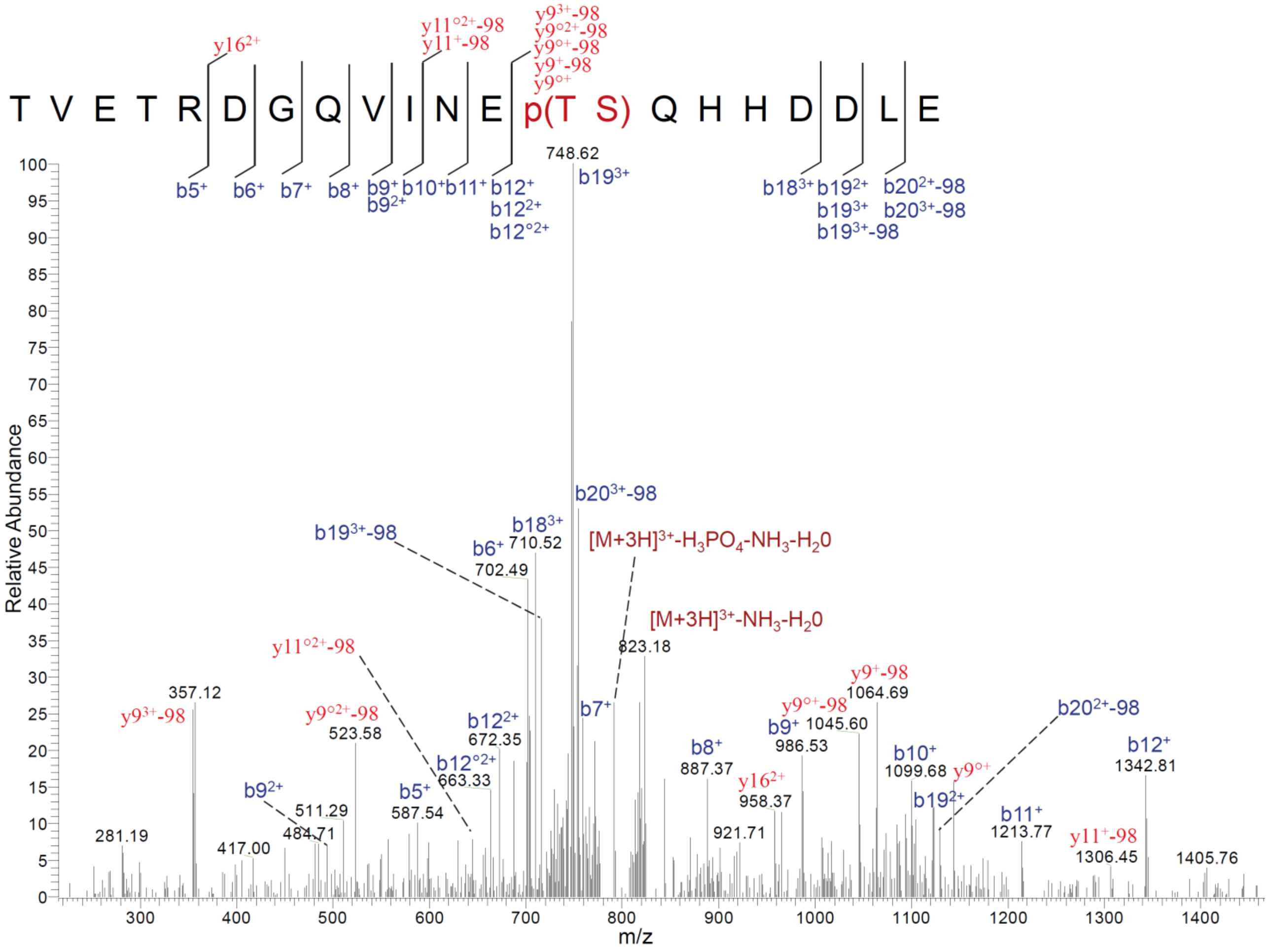

FIGURE S3. MS/MS spectrum (simultaneous fragmentation of neutral loss product and precursor) for identification of vimentin *in vitro* phosphorylation site Ser459. The fragmentation spectrum derived from tryptic vimentin peptide is shown. The peptide sequence and the observed ions of the phophopeptide (inset) are shown, with their spectrum. The tandem mass spectrum was labeled to show singly and doubly charged b and y ions, as well as ion corresponding to neutral losses of water (o), NH_3(*) and H_3PO_4 group (98Da). Fragment ions indicate a phosphorylation of Ser459 or Thr458, but it is not possible to distinguish between these two sites in this analysis, due to their close proximity.
