# Supplementary material for: DNA-PK Target Identification Reveals Novel Links between DNA Repair Signaling and Cytoskeletal Regulation
Source: PLoS One. 2013 Nov 25;8(11):e80313. doi: 10.1371/journal.pone.0080313 (PMC3840018; doi:10.1371/journal.pone.0080313)
Supplement: Figure S4 — MS/MS spectrum for identification of vimentin in vitro phosphorylation site Ser56. (PDF) [file pone.0080313.s004.pdf]

FIGURE S4

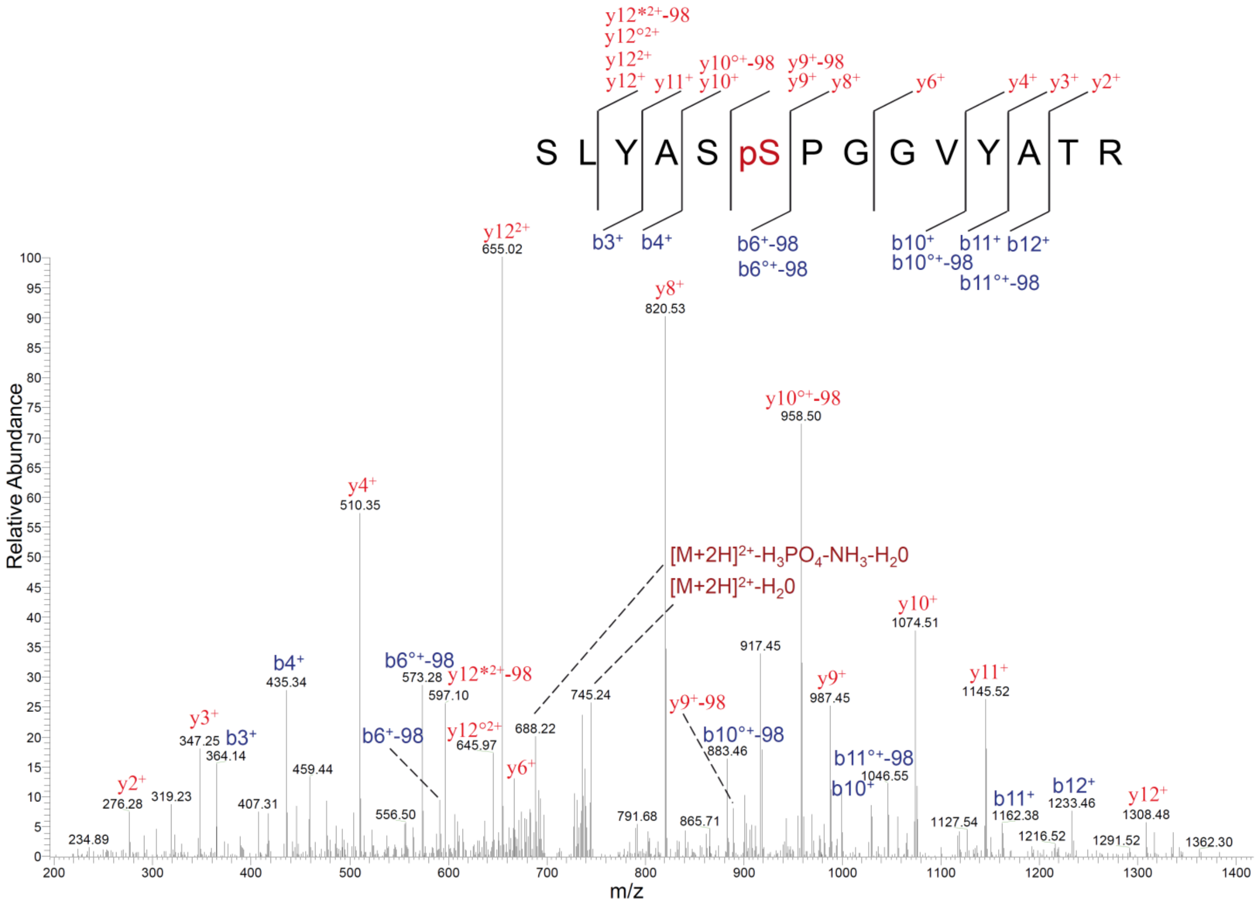

FIGURE S4. MS/MS spectrum (simultaneous fragmentation of neutral loss product and precursor) for identification of vimentin *in vitro* phosphorylation site Ser56. The fragmentation spectrum derived from tryptic vimentin peptide is shown. The peptide sequence and the observed ions of the phophopeptide (inset) are shown, with their spectrum. The tandem mass spectrum was labeled to show singly and doubly charged b and y ions, as well as ion corresponding to neutral losses of water (o), NH_3(*) and H_3PO_4 group (98Da). Fragment ions indicate a phosphorylation of Ser56.
