# Supplementary material for: DNA-PK Target Identification Reveals Novel Links between DNA Repair Signaling and Cytoskeletal Regulation
Source: PLoS One. 2013 Nov 25;8(11):e80313. doi: 10.1371/journal.pone.0080313 (PMC3840018; doi:10.1371/journal.pone.0080313)
Supplement: Table S1 — Detailed MS data including the Mascot Scores of the proteins identified in the 2D gel experiment. (PDF) [file pone.0080313.s006.pdf]

Table S1: Identified proteins phosphorylated upon DNA-PK activation with MS results.

Protein	*Gene name*	*Accession (SwissProt)*	*Identifier (NCBI)*	*MW (Da)*	*Peptides*	*Mascot Score*	*Coverage (%)*	localization[a]
14-3-3 protein τ	YWHAQ	P27348	gi71042776	29197.9	8	474.97	31.2	cytoplasm
14-3-3 protein ζ/δ	YWHAZ	P63104	gi30354619	35335.7	10	742.18	36.9	cytoplasm
DA41	UBQLN1	Q9UMX0	gi12060171	62443.7	5	329.6	15.4	cytoplasm/nucleus
Desmoplakin isoform I	DSP	P15924	gi58530840	331774.0	20	1362.26	10.3	cytoplasm
Eukaryotic translation initiation factor 4A isoform 1	EIF4A1	P60842	gi4503529	46153.9	11	666.79	31.8	cytoplasm
FK506-binding protein 4	FKBP4	Q02790	gi4503729	51804.6	5	355.16	14.4	cytoplasm/nucleus
Heat shock 105kD	HSPH1	Q5TBM3	gi42544159	96865.0	12	937.19	18.8	cytoplasm/nucleus
Heat shock 70kDa protein 1A variant	HSPA1A	P08107	gi62089222	77495.7	15	1104.7	30.2	cytoplasm
Heat shock 70kDa protein 4	HSPA4	Q9BUK9	gi38327039	94331.0	19	1449.16	29.9	N/A
Heat shock 70kDa protein 8 isoform 1	HSPA8	Q53GZ6	gi5729877	70898.1	27	2023.95	45.8	N/A
Heat shock protein 90kDa α (cytosolic), class A member 1 isoform 1	HSP90AA1	P07900	gi153792590	98161.2	2	158.18	3.0	cytoplasm
Histone-binding protein RBBP7	RBBP7	Q16576	gi4506439	47820.1	4	261.29	10.8	nucleus
HMGCS1 protein	HMGCS1	Q01581	gi33991031	61067.8	8	608.71	14.8	cytoplasm
HnRNP F protein	HNRNPF	P52597	gi16876910	45699.9	5	439.39	16.1	nucleus
Lamin B1	LMNB1	P20700	gi15126742	66407.4	12	943.15	21.7	cytoplasm
Myosin, heavy polypeptide 9, non-muscle	MYH9	P35579	gi12667788	226532.0	7	460.05	5.1	cytoplasm/nucleus
Mannose 6 phosphate receptor binding protein 1 (Perilipin-3)	PLIN3	O60664	gi20127486	47032.9	9	743.54	30.9	cytoplasm
NSFL1 (p97) cofactor (p47)	NSFL1C	Q9UNZ2	gi119631035	44621.0	6	395.35	22.0	nucleus
Nucleophosmin/ Nucleoplasmin 3	NPM3	O75607	gi5801867	19305.7	1	101.27	9.0	nucleus
Ribosomal protein SA	RPSA	P08865	gi250127	32767.1	8	611.93	27.5	cytoplasm/nucleus
Tetratricopeptide repeat domain 1[b]	TTC1	Q99614	gi12654245	33525.2	2	94.57	7.5	N/A
Tubulin, β	TUBB	P07437	gi18088719	49671.8	18	1219.48	36.3	cytoplasm/nucleus
Tubulin, β 2C	TUBB2C	P68371	gi20809886	49808.0	19	1343.2	36.6	cytoplasm
Tubulin, β 6	TUBB6	Q9BUF5	gi27754056	50090.4	9	576.46	36.6	cytoplasm
Valosin-containing protein	VCP	P55072	gi111305821	89343.9	15	1023.33	23.4	cytoplasm/nucleus
Vimentin	VIM	P08670	gi340219	53713.7	22	1397.2	47.2	cytoplasm

[a]cytoplasmic or nuclear localization according to UniProtKB (www.uniprot.org)

[b]displayed loss of phosphorylation in response to Dbait 32Hc treatment.
